# Characterization of Decellularized Human Pericardium for Tissue Engineering and Regenerative Medicine Applications

**DOI:** 10.5935/abc.20190094

**Published:** 2019-07

**Authors:** Luciana Wollmann, Paula Suss, João Mendonça, Cesar Luzia, Andressa Schittini, George Willian Xavier da Rosa, Francisco Costa, Felipe F. Tuon

**Affiliations:** Pontifícia Universidade Católica do Paraná, Curitiba, PR - Brazil

**Keywords:** Pericardium, Tissue Banks, Tissue Engineering/trends, Cell Separation, Glycosaminoglycans

## Abstract

**Background:**

Pericardium tissue allograft can be used for surgical repair in several procedures. One of the tissue engineering strategies is the process of decellularization. This process decreases immunogenic response, but it may modify the natural extracellular matrix composition and behavior.

**Objective:**

The aim of this study was to evaluate the effectiveness of cell removal, maintenance of extracellular matrix properties and mechanical integrity of decellularized human pericardium using a low concentration solution of sodium dodecyl sulfate.

**Methods:**

Decellularization was performed with sodium dodecyl sulfate and ethylenediaminetetraacetic acid. Histological analysis, DNA quantification, evaluation of glycosaminoglycans and collagen were performed. Biomechanical assay was performed using tensile test to compare the decellularization effects on tissue properties of tensile strength, elongation and elastic modulus. P < 0.05 was considered significant.

**Results:**

There was reduction in visible nuclei present in pericardium tissue after decellularization, but it retained collagen and elastin bundles similar to fresh pericardium. The DNA contents of the decellularized pericardium were significantly reduced to less than 511.23 ± 120.4 ng per mg of dry weight (p < 0.001). The biomechanical assay showed no significant difference for fresh or decellularized tissue.

**Conclusion:**

The decellularization process reduces cell content as well as extracellular matrix components without changing its biomechanical properties.

## Introduction

Development of extracellular matrix-derived bioscaffolds has been highly desired for applications in tissue engineering and regenerative medicine.^1^ These scaffolds can be obtained from a variety of allogeneic or xenogeneic tissue sources and from several different species.^2^ However, biomaterial antigenicity represents the primary barrier to expanding the use of xenogeneic tissues in clinical practice.^3^

Pericardium is a collagen-rich biological tissue containing glycoproteins and glycosaminoglycans^3^ and is readily available, easy to handle, and pliable. Human pericardium can be used as a patch for heart surgeries^4^ and non-heart surgeries (Peyronie's disease,^4^ glaucoma and corneal surgery,^5,6^ to cover exposed scleral buckles^7^ and oculoplastic surgery.^8^

Human pericardium patch is a well-recognized material for cardiovascular repair.^4^ The physicochemical features of autologous pericardium used for repair include fresh pericardium^9,10^ and glutaraldehyde-treated pericardium.^11^ However, the use of fresh autologous untreated pericardium can result in tissue retraction, thickening, fibrosis and loss of pliability^12^ and the use of glutaraldehyde-treated autologous pericardium can result in calcification.^13^

The main obstacle is the development of biocompatible and functional extracellular matrix-derived bioscaffold. The use of biological tissues increases the potential risks of pathogen transmission^14^ and inflammatory or immunogenic response.^2,15^ Decellularization techniques have been used to minimize these issues.^16^

Tissue decellularization can be performed by using different protocols, detergent extraction or enzymatic extraction with hypotonic or hypertonic washings and physical (agitation, sonication, mechanical pressure or freeze-thawing) treatments.^2^ The ideal decellularization method must remove all antigenic components (nucleic acids, cell membranes, cytoplasmic structures, lipids and soluble matrix) from the tissue without damaging extracellular matrix structure and integrity.^17,18^ However, the decellularization process can modify the natural extracellular matrix composition, as well as mechanical and structural characteristics.^19^ Furthermore, high levels of donor variability to naive ECM composition undoubtedly lead to different ECM fractional composition post-decellularization. Hence, ECM standardization as a biomaterial remains elusive.^18^ Several procedures of decellularization has been described, but this study evaluated low concentration of detergent.

The aim of this study was to evaluate the effectiveness of cell removal, maintenance of extracellular matrix properties and mechanical integrity of decellularized human pericardium using a low concentration solution of sodium dodecyl sulfate.

## Methods

All procedures followed the Brazilian National Regulations and Guidelines according to Regulatory Ordinance nº 2.600, published on October 21, 2009 and ANVISA Resolution - RDC n° 55, published on December 11, 2015. This study was approved by the Ethical Committee of Pontifícia Universidade Católica do Paraná (approval number 1.455.773).

### Donors

Pericardium from 14 non heart-beating and heart-beating donors were obtained by convenience sampling under aseptic conditions. After heart explantation, the organ was placed in a tub with saline solution at 2-8 ºC and the intracavitary blood clots washed. The organ was placed in a sterilized plastic bag, immersed in ice-cold isotonic solution in sufficient quantity to maintain the organ totally immersed. This packaging was sealed, then packaged in two more plastic bags (configuring triple packaging), as each bag must be properly tied or sealed. The triple package containing the organ was placed in an airtight container and placed inside the thermal box in the middle of ice and transported to the human tissue bank (HTB). Dissection was carried out within 48 h after cardiac arrest. Tissue underwent dissection to remove connective tissue and the pericardial sac was cut into strips individually stored in saline solution at 2-8 ºC. Blood samples were tested for HIV, HTLV I/II, HBV, HCV, syphilis, cytomegalovirus (IgM and IgG), toxoplasmosis (IgM and IgG) and Chagas' disease. Donors data were retrieved from their medical records. Positive serology was criteria for rejection except toxoplasma IgG and cytomegalovirus IgG.

### Microbiological control

Before tissue preparation, 30 mL of transport solution (sterile NaCl 0.9% solution) were obtained aseptically in a Class II-A laminar airflow cabinet with a syringe and distributed equally over to a recipient with 90 mL of thioglycolate (Laborclin, Pinhais, Brazil), 90 mL of tryptic soy broth (Laborclin, Pinhais, Brazil) and 90 mL of Sabouraud (Laborclin, Pinhais, Brazil) for bacteriological and mycological testing. The samples were cultured for 14 days at 35°C, 22°C and 22°C, respectively. Cultures were examined daily for visual evidence of turbidity. The ones that showed microorganisms growth were identified for genus and species level.

### Tissue preparation and decontamination

Adherent adipose tissue was dissected with the aid of scissors and forceps. The pericardium was divided into two portions to characterize fresh pericardium versus decellularized pericardium. The pericardia were decontaminated in a RPMI 1640 (Sigma-Aldrich, St. Louis, USA) medium with antibiotics (240 µg/mL cefoxitin, 50 µg/mL vancomycin, 120 µg/mL lincomycin and 100 µg/mL polymyxin B) and were kept between 24 and 48 hours at 2-8°C. Persistent positive cultures after decontamination were used as exclusion criteria.

### Decellularization

Briefly, the pericardia were treated under shaking conditions with a solution of 0.1% sodium dodecyl sulfate (SDS) (w/v) (Sigma-Aldrich, St. Louis, USA) and 7 mM ethylenediaminetetraacetic acid (Sigma-Aldrich, St. Louis, USA) for 24 hours at room temperature. Then, they were washed with 70% ethanol (v/v) for 24 hours at room temperature followed by 10 days of washing with sodium chloride (NaCl) 0.9% solution (Baxter International, Deerfield, USA) to remove residual substances and cellular debris.

### Histological analysis

Longitudinal 3.0 mm cuts with were fixed with neutral buffered formaline 10%, subsequently embedded in paraffin and 4-µm thick sections were taken. Pericardial morphological integrity was analyzed with hematoxylin and eosin (H&E) and Russell-Movat pentachrome (RMP) dyes using an optical microscope BX51 (Olympus Tokyo, Japan). Fresh pericardium was used as control to evaluate changes caused by decellularization. Slides were scanned using an Axio Scan.Z1 slide scanner (Carl Zeiss Microscopy GmbH, Jena, Germany) and subsequent image preparation was performed using software Zen lite (Carl Zeiss, Jena, Germany).

### DNA extraction and quantification

DNA quantification was used to determine the total remaining DNA by comparing the levels in fresh and decellularized pericardium. Tissue samples were weighted, purified and rehydrated using a QIAGEN DNeasy Blood and Tissue Kit (QIAGEN, Valencia, USA) according to the recommended protocol. The concentration of extracted DNA was determined using a Nanodrop spectrophotometer (ThermoFisher Scientific, Wilmington, USA). Extracted DNA sample (1 µL) was loaded onto the Nanodrop and the absorbance was determined at 260 nm. Four readings were taken for each sample and the mean was considered as the absorbance of the sample. DNA concentration was calculated and expressed as micrograms per milligram of dry tissue.

### Biochemical Characterization

#### Sulfated Glycosaminoglycans (sGAG)

The pericardium was digested with papain solution at 60°C for 6 h. Papain (Sigma, St. Louis, MO) was dissolved at 400 mg/mL in 0.1 M phosphate buffer (pH 6.0) with 5 mM cysteine hydrochloride and 5 mM EDTA. The lysates were used for detection of the sGAG amount. The amount of sGAG was measured using a Blyscan sGAG assay kit (Biocolor, Newtownabbey, UK) according to the manufacturer's manual. The tissue lysate was mixed with Blyscan dye to bind the GAG. The GAG-dye complex was then collected by centrifugation. Subsequently, the supernatant was removed and the tube drained, and the dissociation reagent was added. Then solution was transferred into a 96-well plate. Absorbance against the background control was obtained at a wavelength of 656 nm on a Versamax spectrophotometer (Molecular Devices, Sunnyvale, USA). The sGAG amount was calculated based on a standard curve obtained with the standard sGAG supplied with the kit.

#### Collagen

Collagen contents in the pericardium were determined using the Sircol collagen assay (Biocolor, Newtownabbey, UK) according to the manufacturer's instructions. The pericardia were incubated for 48 h at 48°C in 0.5 M acetic acid containing 0.1 mg/ml pepsin. The samples were added to Sircol dye reagent, and collagen-dye complexes formed and precipitated out from the soluble unbound dye. After centrifugation, the pellet was washed once with Acid-Salt Wash Reagent and suspended in alkali reagent. The solution was transferred to a 96-well plate and the absorbance was read at 550 nm on a Versamax spectrophotometer (Molecular Devices, Sunnyvale, USA). The amount of collagen was calculated based on a standard curve obtained with standard bovine type I collagen supplied with the kit.

#### Cytotoxicity Assay

Cytotoxicity of decellularized pericardium was performed in accordance with ISO 10993/5. L929 fibroblasts (ATCC cell line CCL 1, NCTC clone 929) were seeded in 6-well plates (Nest) at a density of 1x10^5^ cells/well. Each well, with inner diameter of 35 mm, received 4 mL of DMEM + 2mM Glutamine + 10% Fetal Bovine Serum. The plates were incubated for 48 h at 37ºC in an atmosphere of 5% ± 0.5% CO_2_ in the air, until a monolayer, with greater than 80% confluence. The medium was removed from the wells and replaced with agar 1.8% added to neutral red dye 0.01%. Samples in triplicate, positive, negative and blank controls on the agar overlay featured 6-well plates. After an incubation period of 24 h at 37 ºC in a humidified atmosphere of 5% ± 0.5% CO_2_ in air, the inhibition zone around the samples was measured, as neutral red is free from dead cells. The biological reactivity of the samples was scored from 0 to 4 in accordance with the following criteria: 0 = absent cytotoxicity (absence of a zone of lysis underneath the sample); 1 = slight (zones of cell lysis underneath the sample only); 2 = mild (zones of cell lysis ≤5 mm from the sample); 3 = moderate (zones of cell lysis>5 mm and ≤10 mm from the sample); 4 = severe (zones of cell lysis >10 mm, but not involving the entire well). In addition, the wells were examined with an inverted microscope (Nikon Eclipse TS 100; Nikon) to observe cell changes.

#### Biomechanical Assay

The biomechanical assay of human pericardium was performed with tensile test to compare the decellularization effects on tissue properties of tensile strength, elongation and elastic modulus. An EMIC DL 500 universal testing machine was used to perform the tests. The sample quantity of fresh and decellularized tissue used was 8 and 6 respectively, and collagen fibers traction direction was random. The dumbbell-shaped sample was based on the ASTM D1708-13 standard because of the small-size tissue available to the tests. To obtain the samples, it was necessary to manufacture a steel rule die according to standard specifications, which was used to cut the pericardium. Before each test, sample thickness, width and initial length values were provided to the equipment. Thickness and width are necessary to calculate the cross-sectional area and consequently the stress (equation 1). Initial length is necessary to calculate the strain and consequently the elongation percentage (equation 2). Thickness was measured with a thickness gauge at three points of the tissue. Pre-load of 0.1 N and 5 mm/min velocity were applied to the test.

**Equation 1. Stress calculation.**

σ=F/A

where:

σ is the stress in megapascal [MPa];

F is the force in newton [N] and

A is the cross-sectional area in square millimeter [mm^2^].

**Equation 2. Calculation of elongation percentage.**

$$\%\mathrm{El}=\left(∆L/\mathrm{Li}\right)\times 100=\varepsilon \times 100$$

where:

%El is the elongation percentage;

ΔL is the variation of sample length in millimeters [mm];

Li is the initial sample length in millimeters [mm] and

ε is the strain in millimeters per millimeter [mm/mm].

### Statistical analyses

Continuous variables with normal distribution were expressed as mean ± SD and median and interquartile range for non-normal distribution, while categorical variables were expressed as frequencies or percentages. Mann-Whitney was used for non-normal distribution of continuous variables, t-test for normal distribution of continuous variables. All tests were two-tailed, and p value < 0.05 was considered significant. Statistical analyses were carried out using SPSS 23.0.

## Results

### Donors' characteristics

The mean age was 39 ± 14 years ranging between 17 and 59 years old. Regarding race, among the samples used, there was a preponderance of donors declared white, 86% (n = 12) of the total and 7% (n = 1) declared themselves as Afro-descendants. An additional 7% (n = 1) was not recorded in the forms.

### Microbiological assessment

Transport solution was positive in 5 samples from 14. *Corynebacterium* spp., *Streptococcus salivarius, Staphylococcus aureus, Klebsiella oxytoca, Anaerococcus prevotii* and *Leuconostoc pseudomesenteroides* were the bacteria identified. Other microbiological tests after decontamination and decellularization were negative.

### Analysis of extracellular matrix integrity and cell removal

**Figure 1:**
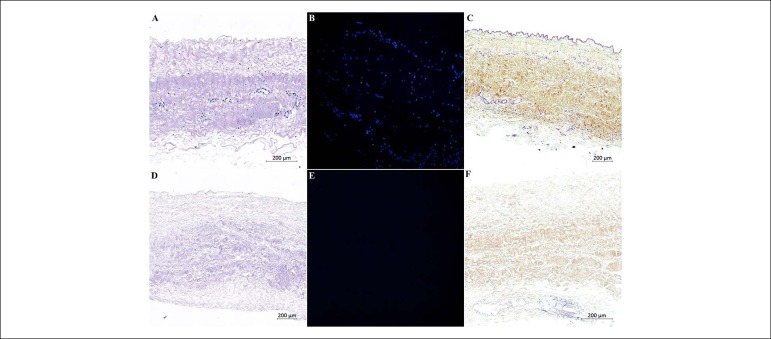
Histological characterization of fresh and decellularized human pericardium. H&E and DAPI staining showed no evidence of cell in the tissue sections (D and E). H&E and RPM staining showed that decellularization did not affect the structure of collagen bundles. Howevers there was a reduction of collagen and increased thickness of the decellularized tissue compared with the fresh one (D and F).

H&E and DAPI staining revealed a reduction in visible nuclei present in pericardium tissue after decellularization (Figure 1A, B, D and E). The main extracellular matrix components were examined in RMP staining (collagen - yellow, elastin - black, and glycosaminoglycans - blue). The structure of the decellularized pericardium extracellular fibers retained collagen and elastin bundles similar to fresh pericardium. However, there was increased thickness of the decellularized tissue compared with the fresh one (Figure 1 C and F).

### DNA quantification

Fresh pericardium tissue with complete cellular contents had a DNA amount as high as 1591±726 ng per mg dry weight. The DNA contents of the decellularized pericardium were significantly reduced to less than 511.23±120.4 ng per mg dry weight. (p < 0.001) (table 1).

**Table 1:** Collagen, glycosaminoglycan and DNA content of fresh and decellularized human pericardium

	Collagen µg/mg	Glycosaminoglycan µg/mg	DNA ng/mg
Fresh pericardium	126.9 ± 45.8	0.64 ± 0.50	1591 ± 726.0
Decellularized pericardium	138.3 ± 58.6	0.02 ± 0.06	511.23 ± 120.4
P value	0.716	0.014	< 0.001

### Effect of decellularization on sulfated glycosaminoglycan and collagen content

The concentration of collagen per mg of dry weight of human pericardium tissue before and after decellularization was 126.9 ± 45.8 and 138.3 ± 58.6 µg/mg of dry tissue, respectively (p = 0.716). Quantification of collagen content in fresh pericardium demonstrated a small increase in the collagen content of the tissue after decellularization. sGAG content was determined to be 0.64±0.50 and 0.02 ± 0.06 sGAG per mg of dry tissue, respectively. (p = 0.014)

### Cytotoxicity assay

**Figure 2:**
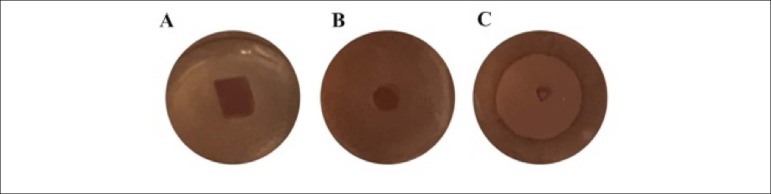
Agar overlay cytotoxicity assay. Testing image after 24 hours in contact with agar-overlay. Decellularized human pericardium (A), negative control (B) and positive control (C).

Decellularized human pericardium (n = 3) was incubated for 24 h (Figure 2A). The samples from the decellularized pericardium had no effect on the cells, as indicated by their normal cell morphology after incubation for 24 h. The negative control also had no effect on the cells (Figure 2B). In contrast, latex (positive control) caused cell death (Figure 2C).

### Biomechanical Assay

**Figure 3:**
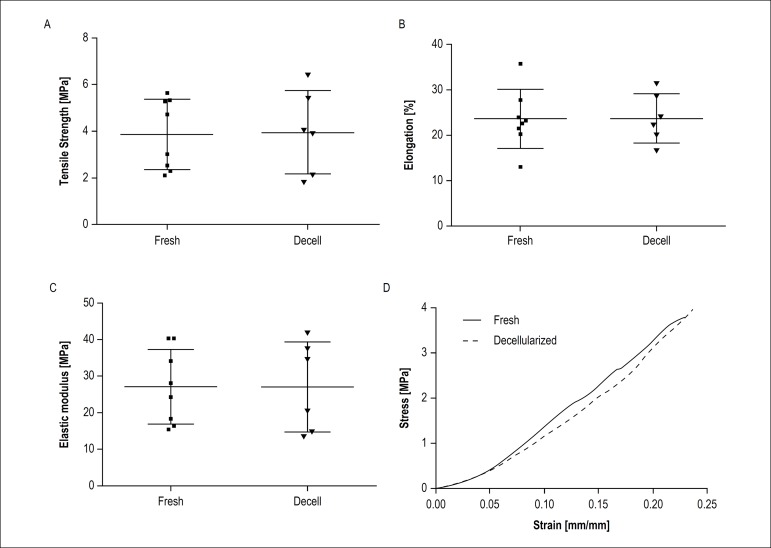
Graphical representation of the mechanical parameters obtained for fresh and decellularized human pericardium. (A) Tensile strength [MPa]; (B) elongation [%]; (C) elastic Modulus [MPa] and (D) the curves indicate the mean stress-strain behavior of fresh and decellularized human pericardium

Average human pericardium thickness showed no significant difference (Welch's t test: p = 0.9518), which was 0.45 ± 0.06 mm for fresh tissue and 0.44 ± 0.05 mm for the decellularized tissue. Tensile strength, elongation and elastic modulus are presented as column scatter plot graphs (Figure 3A, B and C) showing the mean and standard deviation. The mean difference among the groups was not significant for all properties (p > 0.05). The average stress-strain curve of fresh and decellularized tissue are presented in Figure 3D, which shows the typical behavior for biological tissues as previously described.^20^ It is also possible to observe that the behavior of curves was similar in the initial phase until transition phase, keeping approximate parallels until maximum stress.

## Discussion

The aim of decellularization is to minimize the immune response by completely removing cellular components as well as preserving the physical characteristics of the extracellular matrix.^2,21^ The ideal decellularized tissue must achieve complete decellularization and preservation of the extracellular matrix for biomechanical stability and function, but it is recognized that any decellularization protocol may result in extracellular matrix disruption and potential loss of surface structure and composition.^2^

In the histological analysis, this study revealed that most cellular components were removed, but some components of the extracellular matrix were reduced and the decellularized tissue thickness increased. Similar findings were reported by Courtman et al.^22^ with a 3-fold increase in tissue thickness. However, Mirsadraee et al.^23^ did not observe any significant changes using an SDS-based decellularization protocol in the histological analysis comparing both fresh and decellularized human pericardium.

In this study, decellularized human pericardium got a small increase in the collagen content and a significant reduction in sGAG content. In agreement with our observation, Mendoza-Novelo et al.^24^ reported a reduction in fresh pericardium tissue sGAG content after decellularization with surfactant tridecyl alcohol ethoxylate and reversible alkaline swelling.^24^ On the other hand, a study by Mirsadraee et al.^23^ reported a small increase in hydroxyproline and GAG content in the tissue after decellularization.^23^ The authors concluded that increase of hydroxyproline was due to a relative increase in the ratio of these molecules regarding total dry weight, due to the loss of soluble proteins and cell components.

In order to ensure adequate residual removal, contact cytotoxicity assays were performed to determine the effect of any chemical residuals in the decellularized tissue on the growth of cultured L929 cells. Assay results showed that cells grew well in contact with samples of decellularized human pericardium, indicating that the decellularized tissues were non-toxic. This result also correlates with previous study on decellularized human pericardium.^23^

We compared the mechanical properties of fresh and decellularized pericardium and there were no significant differences between them. SDS-based decellularization by itself does not modify the performance of human pericardium as for the parameters of tensile strength, elongation and elastic modulus. These results were previously assessed in the acellular human pericardium.^23^ In a more recent work, the biomechanical properties of decellularized and cryopreserved human pericardium were preserved in comparison with fresh tissue.^25^

Nevertheless, this study did not evaluate the outcome of implanted human decellularized pericardium. In vitro analysis is not enough to confirm complete decellularization. The maintenance of extracellular matrix in this study does not allow to determine that all molecular and physiological properties are really ideal.

## Conclusion

The decellularization process reduces cell content as well as extracellular matrix components and increases thickness without changing the biomechanical properties of the decellularized tissue compared with the fresh one. Decellularized human pericardium may be a suitable candidate for the production of extracellular matrix-derived scaffolds for tissue engineering and regenerative medicine applications.
